# Editorial: The effect of anti-cancer drug therapies in the treatment of lung cancer

**DOI:** 10.3389/fonc.2022.1035245

**Published:** 2022-10-19

**Authors:** Pasquale Pisapia, Zhi Li, Cyril Corbet

**Affiliations:** ^1^ Department of Public Health, University of Naples Federico II, Naples, Italy; ^2^ Department of Medical Oncology, The First Affiliated Hospital of China Medical University, Shenyang, China; ^3^ Institut de Recherche Expérimentale et Clinique (IREC), Pole of Pharmacology and Therapeutics, Brussels, Belgium

**Keywords:** lung cancer, NSCLC, molecular, target therapy, biomarker

Lung cancer is still the leading cause of cancer-related deaths worldwide (1). Patients with lung cancer feature poor clinical outcomes primarily due to the challenges of early detection, high risk of metastasis and the development of resistance to multiple therapies. Thus, a deeper understanding of the molecular mechanisms supporting therapy escape is essential to improve the survival rate and prognosis of this disease. Currently, international guidelines have established a minimum panel of so-called “must test genes”, including epidermal growth factor receptor (*EGFR*), Kirsten Rat Sarcoma Viral Oncogene Homolog (*KRAS*) and V-Raf Murine Sarcoma Viral Oncogene Homolog B (*BRAF*) mutations, Anaplastic Lymphoma Receptor Tyrosine Kinase (*ALK*), ROS Proto-Oncogene 1, Receptor Tyrosine Kinase (*ROS1*), Rearranged During Transfection (*RET*), Neurotrophic Receptor Tyrosine Kinase (*NTRK*) gene rearrangements and MET Proto-Oncogene, Receptor Tyrosine Kinase (*MET*) exon 14 skipping, for tyrosine kinase inhibitors (TKIs) administration and the evaluation of the expression level of Programmed death-ligand 1 (PD-L1), for immune-checkpoint inhibitors (ICIs) administration, in order to avoid to leave any non-small cell lung cancer (NSCLC) patient behind (2). Beyond well-established biomarkers, others are being currently under investigation as potentially actionable genomic alterations. (Zhou et al.; Zhai et al.)

Overall, in this Special Topic of Frontiers in Oncology and Frontiers in Pharmacology, we attempt to address some major concerns related to targeted therapies and ICIs administration, novel therapeutic strategies, resistance mechanisms and adverse events in lung cancer patients.

The results of FLAURA clinical trial have clearly demonstrated the superiority in terms of clinical benefit of first-line osimertinib respect to other EGFR TKIs in untreated advanced stage NSCLC patients harboring common (exon 19 deletions or exon 21 p.L858R) *EGFR* mutations (3, 4). However, little was known about the efficacy of this drug on uncommon *EGFR* mutant patients. In this setting, Zhi et al., in a case report experience, highlighted that the combination of osimertinib plus bevacizumab may represent a valid therapeutic option after osimertinib in advanced stage NSCLC patients harboring *EGFR* exon 20 p.D770_N771insGT mutation and brain metastasis.The combination of osimertinib plus bevacizumab has also demonstrated a higher clinical efficacy respect to chemotherapy plus bevacizumab in advanced stage NSCLC *EGFR*-mutant patients after the failure of osimertinib alone administration (Cui et al.). In addition, osimertinib seemed to be safe and feasible also in neoadjuvant setting in resectable NSCLC *EGFR*-mutant patients (Hu et al.). A crucial point in the management of advanced stage NSCLC patients by EGFR TKIs is represented by the possibility to act on brain metastasis. As for osimertinib, almonertinib, a novel third generation EGFR TKI, showed in preclinical models the capacity to easily penetrate the blood-brain barrier and the ability to act on brain and spinal cord metastases (Zhang et al.). Beyond *EGFR*-mutant patients, careful attention should be paid on gene rearrangements. In this setting, Peng et al. highlighted, in a Bayesian network meta-analysis, that lorlatinib is associated with the highest progression-free survival (PFS) and lowest risk of brain progression in advanced stage NSCLC *ALK*-fused patients respect to other ALK TKIs. However, a significant higher toxicity was also registered. Regarding small cell lung cancer and other rare large cell neuroendocrine lung carcinomas, encouraging results have been reported with the adoption of the novel multi-targeting TKI anlotinib (Gan et al.; Xu et al.). Regarding ICIs administration, beyond the established role of expression level of PD-L1, other biomarkers are being under investigation. Among these, Fu et al. showed the benefit of ICI administration in an advanced stage NSCLC patients with DNA Polymerase Epsilon, Catalytic Subunit (*POLE*) mutation and brain metastasis.

The role of POLE2 was also highlighted in the study by Ni et al. In this work, the authors showed that berberine, a natural anti-cancer drug, may inhibit *in vitro* and *in vivo* lung adenocarcinoma progression due to the down-regulation of Forkhead Box M1 (*FOXM1*)/*POLE2* expression as emerged.Among the novel therapeutic strategies, several other molecules are being currently under investigation and are showing promising pre-clinical results. (Pan et al.; Wang et al.; Wu et al; Yan et al.; Ye et al.; Yin et al.; Zhang et al.; Zhao et al.; Zhao et al.). As far as chemotherapy is concerned, Tu et al. showed that the inhibition of hedgehog signaling may a be a promising strategy to improve paclitaxel response in advanced stage NSCLC *EGFR* wild type patients.

Another crucial point in lung cancer treatment is represented by the development of resistance. In this scenario, several mechanisms may arise. In the case report by Liu et al., a patient harboring an *EGFR* exon 21 p.L858R point mutation developed a Lysocardiolipin Acyltransferase 1 (*LCLAT1*)/*ALK* rearrangement after icotinib administration and an additional *EGFR* exon 20 p.T790M after the subsequent ensartinib administration. Thus, in those patients an association of osimertinib plus ensartinib has been considered to overcome the double resistance mechanisms.Considering gene rearrangements, *MET* amplification has been identified as a resistance mechanism to crizotinib administration in advanced stage NSCLC *ROS1*-fused patients by Yang et al.

Finally, the role of adverse events to chemotherapy and targeted therapies, including TKIs and ICIs, should be taken into account. Regarding antimetabolites, Geier et al. described the development of a severe acrosyndrome. The authors suggested awareness for the rise of these events, in particular in patients with predisposing factors. As far as TKI regimen is concerned, a rare and severe fatal tumor lysis syndrome has been showed after brigatinib administration in an advanced NSCLC *ALK*-fused patients. (Wang et al.). A possible solution to reduce adverse events in afatinib administration has been proposed by Wang et al. The authors in their systematic review and meta-analysis highlighted a significant reduction in adverse events with a dose reduction of the drug. Rare adverse events have been also reported for ICI regimens. In particular, Lin et al. reported a hepatic sarcoid/granulomatous-like reaction after toripalimab (anti PD-1) administration whereas a severe pneumonitis was reported by Xie et al. after pembrolizumab administration. However, in this latter case, the Authors highlighted the role of nintedanib for the treatment of this adverse event (Xie et al.).

Overall, this Research Topic has highlighted the effects, in terms of clinical benefit, resistance mechanisms and adverse events, of different anti-cancer drugs in the treatment of lung cancer. Ongoing research is warranted to improve the clinical outcome of these patients.

## Author contributions

All authors listed have made a substantial, direct, and intellectual contribution to the work and approved it for publication.

## Conflict of interest

PP has received personal fees as speaker bureau from Novartis, for work performed outside of the current study.

The remaining authors declare that the research was conducted in the absence of any commercial or financial relationships that could be construed as a potential conflict of interest.

## Publisher’s note

All claims expressed in this article are solely those of the authors and do not necessarily represent those of their affiliated organizations, or those of the publisher, the editors and the reviewers. Any product that may be evaluated in this article, or claim that may be made by its manufacturer, is not guaranteed or endorsed by the publisher.
